# Oil palm expansion reshapes *Culicoides* assemblages and avian haemosporidian infections

**DOI:** 10.1186/s13071-026-07319-y

**Published:** 2026-03-10

**Authors:** Rafael Gutiérrez-López, Bruno Mathieu, Boris K. Makanga, Christophe Paupy, Nil Rahola, Vincent Bourret, Martim Melo, Claire Loiseau

**Affiliations:** 1 National Center of Microbiology, Health Institute Carlos III., Ctra Pozuelo 28, Majadahonda, 28220 Madrid, Spain; 2 Ciber de Enfermedades Infecciosas (CIBERINFEC), 28050 Madrid, Spain; 3 Institute of Bacteriology and Parasitology, Medical Faculty, University of Strasbourg, UR 3073 PHAVI, 67000 Strasbourg, France; 4 Institut de Recherche en Écologie Tropicale/CENAREST, BP 13354 Libreville, Gabon; 5 MIVEGEC, Montpellier University, IRD, CNRS, 34394 Montpellier, France; 6 CEFS, INRAE, Toulouse, France; 7 CIBIO-InBIO - Research Center in Biodiversity and Genetic Resources, InBIO Associate Laboratory, Campus de Vairão, 4485-661 Vairão, Portugal; 8 MHNC-UP, Natural History and Science Museum of the University of Porto, Porto, Portugal; 9 FitzPatrick Institute of African Ornithology, Department of Biological Sciences, University of Cape Town, Rondebosch, South Africa

**Keywords:** Blood meal, *Biting midges*, Dilution effect, Haemosporidians, Monoculture

## Abstract

**Background:**

Land-use change can influence parasite transmission by reshaping ecological interactions among parasites, vectors, and hosts. In particular, deforestation and agricultural expansion modify habitat structure and resource availability, potentially altering the prevalence and distribution of vector-borne diseases.

**Methods:**

Fieldwork was conducted on São Tomé Island (Gulf of Guinea, Central Africa) across a land-use gradient from the core of an oil palm plantation to adjacent native forest. *Culicoides* biting midges and birds were sampled across four habitat types (village, oil palm plantation, at the border between the plantation and the forest, and forested areas) using Centers for Disease Control (CDC) traps and mist nets, respectively. DNA extracted from *Culicoides* and bird blood was used to screen for *Plasmodium*, *Haemoproteus*, and *Leucocytozoon* using nested polymerase chain reaction (PCR). Blood-fed *Culicoides* collected in the traps were analyzed by PCR to identify the host species. Linear models were used to assess differences in vector diversity, abundance, host-feeding preferences, and haemosporidian prevalence among habitats.

**Results:**

*Culicoides* species richness did not differ significantly between habitats, but species abundances did vary. Overall abundance was lower in the oil palm plantation than in border and forest areas. Mammophilic *Culicoides* were more abundant in the village, whereas ornithophilic species were predominated in the forest.

We screened 432 bird blood samples and 452 parous *Culicoides* for haemosporidian infections. Haemosporidian parasites were most frequently detected in *Culicoides* pools from the border area. Among birds, *Plasmodium* prevalence was significantly higher in the oil palm plantation than in border and forest habitats, while *Leucocytozoon* infections were completely absent in plantation birds.

**Conclusions:**

Anthropogenic habitat disturbance modifies vector communities and host–parasite associations, influencing the transmission dynamics of *Haemoproteus* parasites. These findings highlight the ecological consequences of agricultural expansion and the importance of preserving natural habitats to mitigate disease emergence under land-use change scenarios.

**Graphical Abstract:**

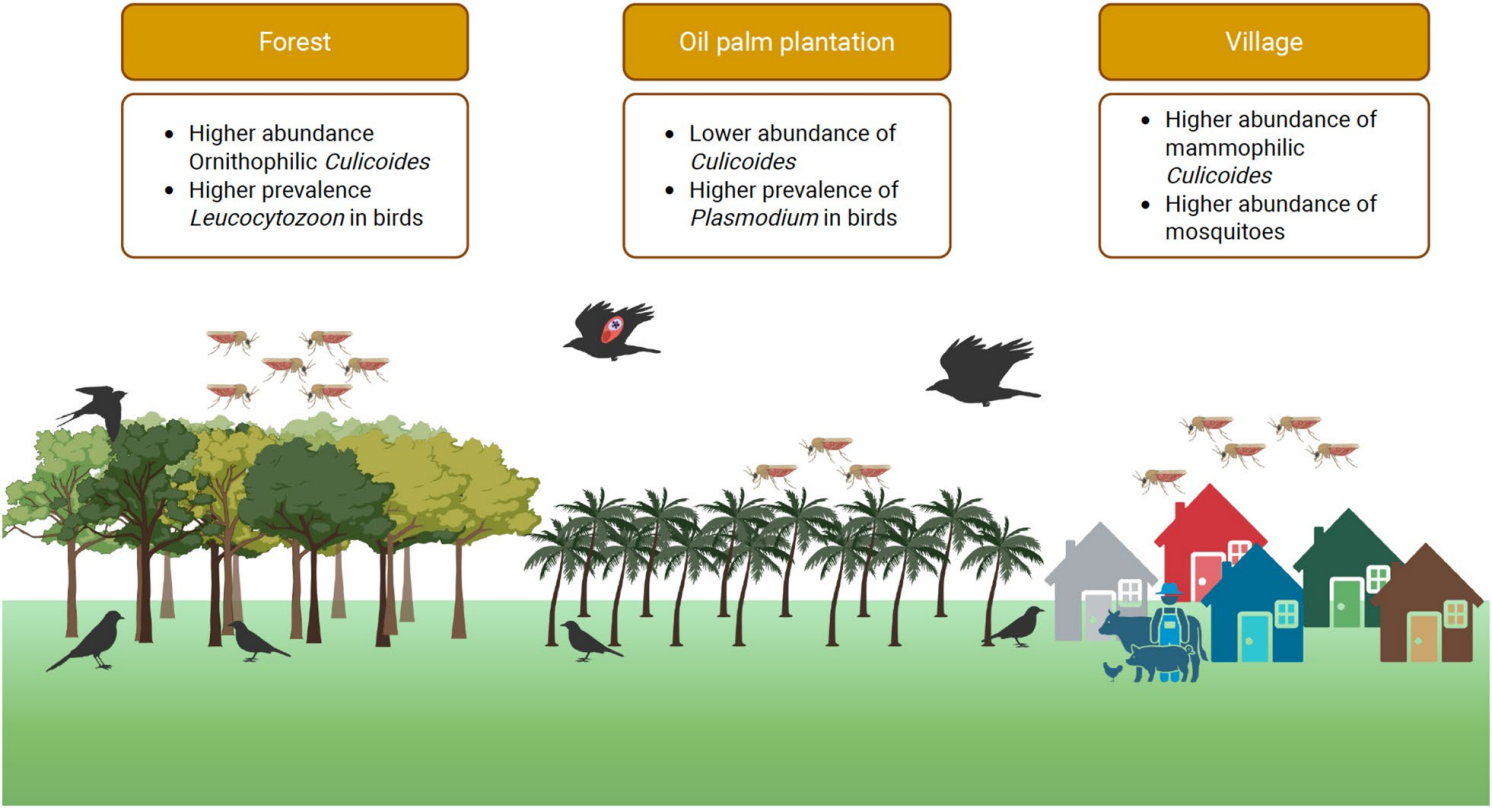

**Supplementary Information:**

The online version contains supplementary material available at 10.1186/s13071-026-07319-y.

## Background

Agroindustry is a major driver of deforestation in tropical regions. Over recent decades, monoculture plantations have expanded globally [1–3]. This expansion has degraded critical ecosystem functions [1] and caused significant biodiversity loss [4] across multiple taxa, including arthropods [5], birds [6], amphibians [7], and mammals [8].

Land-use changes and agricultural practices also indirectly affect pathogen transmission by altering host communities and the abundance of competent reservoirs and vectors [9]. The dilution effect hypothesis suggests that biodiversity loss in disturbed habitats can increase pathogen prevalence [10]. However, this relationship remains debated, as amplification effects may also occur depending on vector ecology and feeding habits [11, 12]. Studies on mosquito-borne diseases illustrate this variability, with transmission reported to increase [13], decrease [14], or remain unchanged [15] in agricultural or urban areas. Factors such as vector diversity [16], vector abundance [17], adaptability to new habitat conditions [18], and feeding behavior [19] strongly influence these outcomes. A notable example is *Plasmodium knowlesi* in Southeast Asia, which became a zoonotic pathogen, transferring from macaques to humans, as forest-dwelling mosquitoes (*Anopheles* from the *Leucosphyrus* group) adapted to fragmented habitats, agricultural lands, and human settlements [20]. Similarly, a study in central Panama found higher abundance of *Culicoides* species in forested areas than in disturbed areas [21]. Several studies have examined the impacts of forest conversion to monocultures, particularly oil palm, on vector populations across the tropics [22–24]. These studies suggest that oil palm plantations may provide an unsuitable environment for mosquito populations, resulting in reduced diversity. For instance, mosquito species were almost absent from the oil palm plantations in Malaysia, except for *Aedes albopictus* [23]. In contrast, research in Côte d'Ivoire reported a higher mosquito genus diversity in adjacent rainforest and residential areas than in plantations [22].

Despite growing knowledge of vector distribution, diversity, and abundance, less is known about how blood-feeding patterns vary along gradients of anthropogenic disturbance, even though these patterns are critical for pathogen transmission. Identifying blood sources using molecular tools applied to engorged specimens can help address this gap [25]. However, capturing blood-fed vectors remains challenging, especially in tropical environments with diverse resting sites [26, 27], and accurate identification of highly diverse tropical vector fauna is often insufficiently documented.

Islands provide ideal systems to investigate pathogen transmission mechanisms, as they experience similar agricultural pressures as mainland regions but generally harbor lower species diversity [28]. This allows interactions among vectors, hosts, and pathogens to be studied in a more controlled context. However, integrative studies simultaneously addressing these components remain rare and require multidisciplinary collaboration among ecologists, ornithologists, parasitologists, and entomologists [29].

Although several studies have explored vector ecology in tropical systems [18, 30], information on vector-borne parasite transmission in São Tomé remains limited. In particular, the diversity and distribution of mosquitoes (Culicidae) and black flies (Simuliidae), vectors of *Plasmodium* and *Leucocytozoon*, respectively, remain poorly known. While infections by *Plasmodium* and *Leucocytozoon* have been reported in birds on the island [31, 32], no studies have identified or quantified the prevalence of their insect vectors, representing a major gap in understanding the avian haemosporidian transmission across habitats on the island. Vector studies have historically focused on mosquitoes [33], but recent work has expanded to biting midges, which transmit pathogens affecting humans, livestock, and wildlife [34]. Notably, several *Culicoides* species are key vectors of *Haemoproteus* parasites responsible for avian haemoproteosis [35]. Previous studies in traditional agroforestry systems on São Tomé, particularly shade-grown cocoa and coffee plantations, have reported infections by *Plasmodium*, *Haemoproteus*, and *Leucocytozoon* in birds [31, 32]. However, these studies focused exclusively on avian hosts and did not assess parasite prevalence in insect vectors. Moreover, no studies have examined the presence of haemosporidian parasites, either in birds or vectors, in industrial plantation systems on the island, such as oil palm monocultures. The avian community in São Tomé’s oil palm plantations is composed of nonnative, habitat-generalist bird species, along with a few endemic species capable of exploiting both forested and plantation habitats [36].

This study investigated tripartite interactions among hosts, vectors, and parasites in a bird–insect vector–avian haemosporidian system across a gradient of anthropogenic disturbance, from the core of an oil palm plantation (including the adjacent village) to native forest. The main objective was to determine how land-use change modulates vector-borne transmission dynamics along this gradient. Specifically, we aimed to (i) quantify the diversity and abundance of insect vectors (mosquitoes, biting midges, and black flies), transmitting *Plasmodium*, *Haemoproteus*, and *Leucocytozoon*, respectively; (ii) characterize vector blood-feeding patterns across habitats; and (iii) assess haemosporidian parasites prevalence in birds and vectors.

## Methods

### Study area

**Fig 1:**
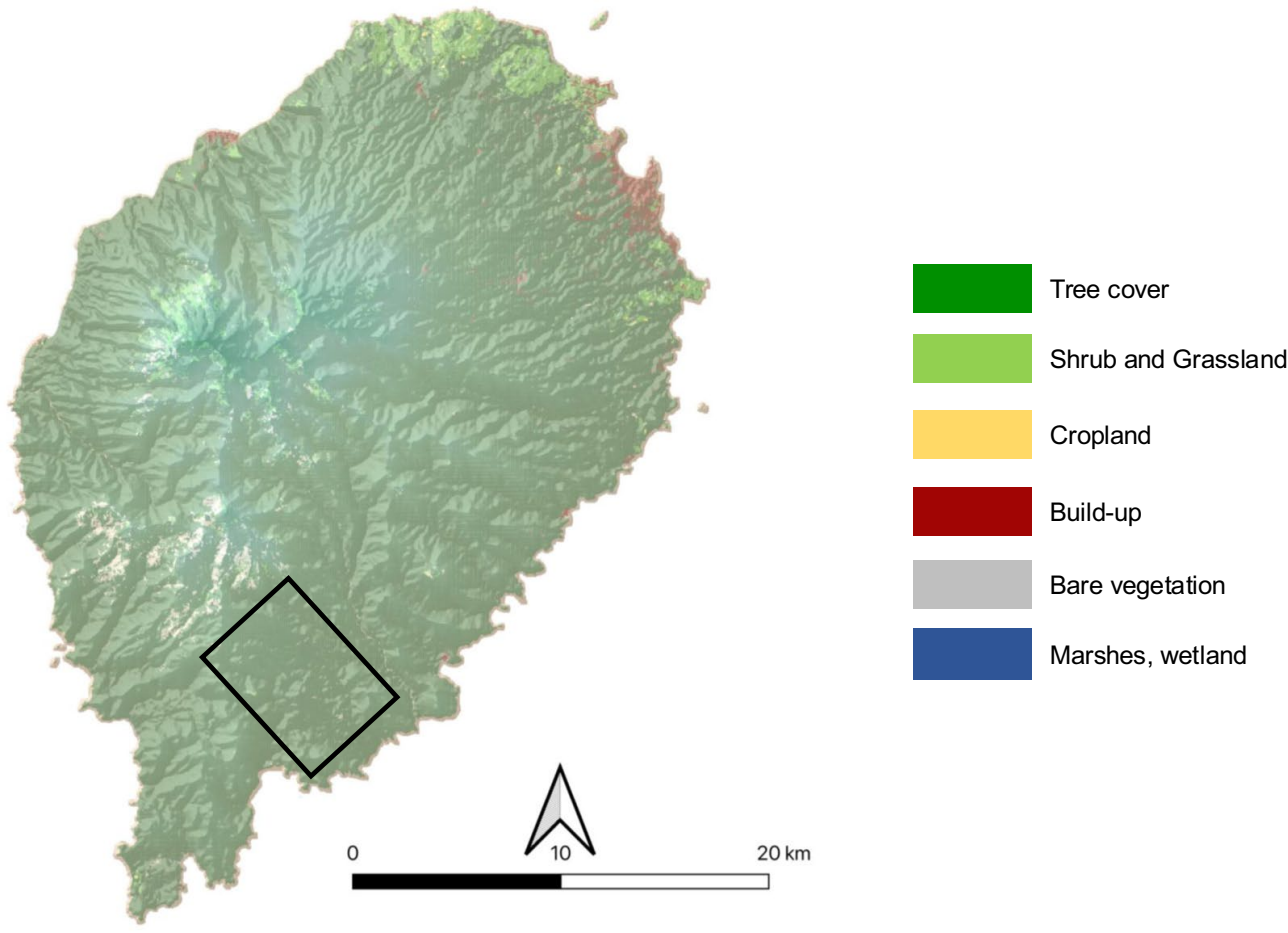
Land uses on São Tomé Island. The black rectangular frame represents the location of the study in the southeast region

São Tomé is a volcanic oceanic island located about 300 km off the northwestern equatorial coast of Gabon (Gulf of Guinea, Central Africa), along the volcanic Cameroon line [37] (Fig. 1). The island shows exceptional endemism across taxa [38], particularly among birds, with 17 single-island endemics, and 3 endemics shared with the neighboring islands of Príncipe and Annobón [39]. The climate is equatorial, with average annual temperatures ranging from 22 °C to 30 °C, and alternating wet and dry seasons. Original vegetation was predominantly tropical rainforest [40]. However, much of the lowland forest has been cleared, with remaining patches mainly restricted to southern regions. In late 2009, an industrial oil palm company acquired a substantial concession close to the Obô Natural Park, in some areas encroaching upon its buffer zone and replacing native forest with oil palm monocultures. This study was conducted in the southeastern part of the island, within a landscape dominated by oil palm plantations (Fig. 1; 0°6′57″ N, 6°35′33″ E).

### Vector sampling

**Fig 2:**
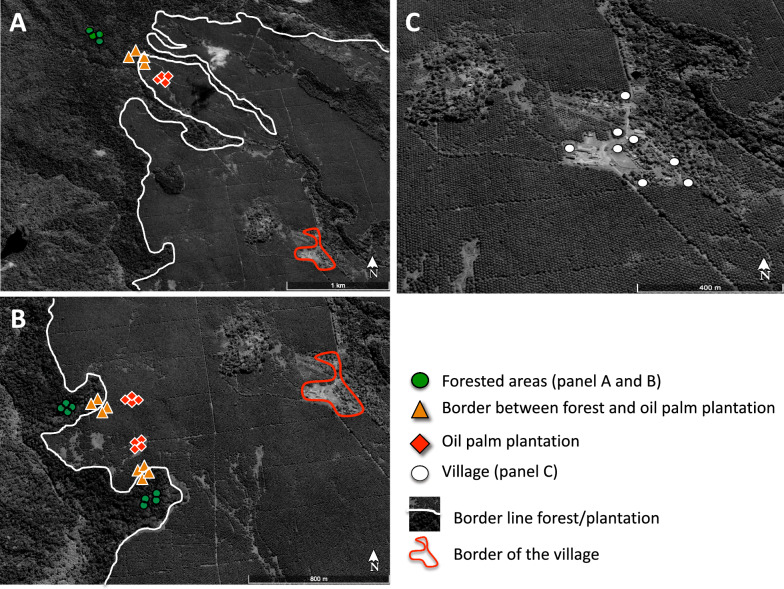
Sampling sites for *Culicoides* spp. on São Tomé Island. Each color symbol represents one light trap along the gradient of anthropogenic disturbance. **A**: north of the plantation with the forest; **B**: west of the plantation with the forest; **C**: village

Vector sampling was conducted in October 2019, at the beginning of the rainy season, across three transects of three habitat types: (i) the oil palm plantation, (ii) the border between the plantation and forest (arising from logging and plantation abandonment), and (iii) forested areas (Fig. 2). Traps were deployed in the afternoon, operated overnight, and samples collected the following morning. Sampling effort was standardized across habitats, with 12 CDC traps placed along the 3 transects from forest to plantation, (i.e., 4 traps per habitat; forest/border/plantation; Fig. 2A and B). Each habitat was sampled for 5 nights, ensuring equal trapping effort across all habitats in terms of number of sites, traps, and CDC trap-hours per habitat. Distances between traps in the forest and the plantation ranged from 600 m to 1 km. Vector sampling was also performed during 3 nights in the village located in the center of the oil palm plantation. In the village, we placed eight traps to cover the variety of habitats (close to the chicken roost, pigs’ pens, human habitation, or near the vegetation on the village border; Fig. 2C).

Diptera collected were sorted by family the next day, focusing on mosquitoes, biting midges, and black flies; however, we failed to collect any black flies. Mosquito species were identified morphologically using published keys [41–45], with nomenclature following the Walter Reed Biosystematics Unit Mosquito Catalogue (http://www.mosquitocatalog.org). For the biting midges, we focused on the genus *Culicoides* identified to species level, back in the laboratory, using morphological characters by dichotomous keys [46, 47].

Due to the exceptionally high abundance of *Culicoides* captured during sampling, processing all individuals from each trap was impractical. Consequently, the total number of sampling tubes (2 ml) retained for laboratory analyses (*n* = 340) was not predefined, but resulted from the total volume of specimens collected per trap (mean number of tubes per trap = 13.1; range 1–20). To ensure a balanced and comparable sampling effort across sites and habitat types, we applied a standardized subsampling strategy. For each trap and habitat, a fixed number of tubes (five) was selected for analysis using a randomization procedure. Specifically, tube selection was performed by generating random numbers, which were used to randomly identify five tubes per trap and habitat. This approach allowed us to estimate *Culicoides* diversity and relative abundance while minimizing biases associated with unequal sample sizes among traps and habitats.

Engorged females were retained for blood-meal analyses, and parous females of each species were pooled for parasite detection. Parous females were identified on the basis of the presence of a burgundy-red pigmented abdomen indicative of a first gonotrophic cycle [48]. Pools were defined by species, habitat, and transect to assess the prevalence of haemosporidian parasites in vectors. Pools ranged from 1 to 10 individuals (Additional file 1: Table S1).

### Bird sampling

**Fig 3:**
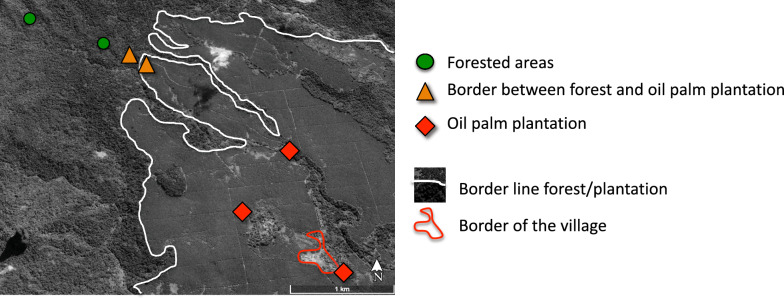
Sampling sites of birds across different habitats on São Tomé Island. In each site 4–6 12-m mist nets were deployed

Birds were captured using mist-nets (Ecotone, Poland) and ringed with SAFRING metal ring during field sessions in May 2016 (rainy season), July 2018 (dry season), and 2019 (rainy season) at seven sites (Additional file 1: Table S2). The sampling sites included: (i) two sites within the oil palm plantation (1 km apart), (ii) two at the border (200 m apart from each other, 100 m from the border) and the forest, and (iii) two sites in the forest (at 800 m apart) (Fig. 3). We also sampled birds at one site located at the edge of the village that we classified as in the plantation site since the nets were placed on the first line of palm trees, and since it was impossible to have replicate sampling site within the village. Blood samples were collected by brachial venipuncture, using capillaries, treated with heparin, and preserved in 96% ethanol until DNA extraction. Following sampling, all birds were released at the capture site. Only adult individuals and resident species were selected for this study.

### Molecular analyses

#### Parasite screening in birds and insect vectors

DNA was extracted from bird blood samples and parous *Culicoides* (whole individuals) using Minikit PureLink™ Genomic DNA (Invitrogen, Waltham, Massachusetts, U.S.) and DNeasy Blood & Tissue Kits (Qiagen, Hilden, Germany), respectively. Few mosquito specimens were collected, which did not allow for reliable results and therefore we did not extract DNA from them. Haemosporidian parasites (*Plasmodium*, *Haemoproteus*, and *Leucocytozoon*), were detected using a nested PCR targeting a 479 bp fragment of the parasite mtDNA, cytochrome* b* gene [49]. The first PCR used primers HaemNFI (5′- CAT ATA TTA AGA GAA ITA TGG AG −3′) and HaemNR3 (5′- ATA GAA AGA TAA GAA ATA CCA TTC −3′), followed by a nested PCR using primers HaemF (5′- ATG GTG CTT TCG ATA TAT GCA TG −3′) and HaemR2 (5′- GCA TTA TCT GGA TGT GAT AAT GGT −3′) for *Haemoproteus* spp. and *Plasmodium* spp., and the primers HaemFL (5′- ATG GTG TTT TAG ATA CTT ACA TT −3′) and HaemR2L for *Leucocytozoon* (5′- CAT TAT CTG GAT GAG ATA ATG GIG C −3′). The first PCR was performed in volume of 25 µl, which included approximately 50 ng of total genomic DNA, 1.25 mM of each deoxynucleoside triphosphate, 1.5 mM MgCl2, 1X PCR (Applied Biosystems, Foster City, California), 0.6 mM of each primer, and 0.5 units Taq DNA polymerase. The PCRs including HaemNFI–HaemNR3 were conducted using the following conditions: 30 s at 94 °C, 30 s at 50 °C, and 45 s at 72 °C for 35 cycles. The samples were incubated before the cyclic reaction at 94 °C for 3 min and after the cyclic reaction at 72 °C for 10 min. We used 2 µl of the first PCR reaction as the template for the second PCR, using the primers (HaemF–HaemR2) for *Haemoproteus* spp. and *Plasmodium* spp., and the primers (HaemFL-HaemR2L) for *Leucocytozoon* spp. These PCRs were performed separately in 25 µl volumes with the same proportions of reagents as in the initial PCR reactions and conditions. Each PCR included a positive control and a negative control. PCR products were visualized on 2% agarose gel and successful amplicons were sequenced by GENEWIZ (Leipzig, Germany). Sequences were edited and aligned using GENEIOUS 7 [50], and parasite lineages compared with reference sequences from GenBank and MalAvi databases [51].

#### Blood meal analyses

Abdomens of blood-fed female *Culicoides* were cut from the head/thorax using sterile tweezers, and genomic DNA was extracted using DNeasy Blood & Tissue Kits (Qiagen, Hilden, Germany). To amplify a 758 bp fragment of the vertebrate mitochondrial cytochrome c oxidase subunit I (COI) gene, we conducted a nested PCR using eukaryote-universal forward and vertebrate-specific reverse primers. The primary PCR was performed using primers BCFW-M13 (5’- TGT AAA ACG ACG GCC AGT HAA YCA YAA RGA YAT YGG −3’) and BCRV1 (5’- GCY CAN ACY ATN CCY ATR TA-3’), followed by a nested PCR using primers M13 (5’- GTA AAA CGA CGG CCA GTG −3’) and BCRV2 (5’- ACY ATN CCY ATR TAN CCR AAN GG −3’). The first PCR was carried out in a final volume of 30 µl containing 1 unit of Taq DNA polymerase (Bioline), 1X manufacturer-supplied buffer, 2.5 mM MgCl₂, 0.25 mM of each deoxynucleoside triphosphate, 5% DMSO, 10 µg of bovine serum albumin (BSA; Amersham Corp.), 0.16 µM of each primer (BCFW-M13 and BCRV1), and 1 µl of extracted DNA. The amplification conditions consisted of an initial denaturation step of 4 min at 94 °C, followed by 35 cycles of 40 s at 94 °C, 40 s at 45 °C, and 1 min at 72 °C, with a final extension step of 7 min at 72 °C. For the nested PCR, 1 µl of the first PCR product was used as template. Reactions were performed in a final volume of 30 µl with the same reagent proportions as the initial PCR, except for the MgCl_2_ concentration (1.7 mM), BSA amount (5 µg), and the use of primers M13 and BCRV2 at a concentration of 0.16 µM each. The nested PCR protocol consisted of an initial denaturation step of 3 min at 94 °C, followed by a touchdown phase decreasing the annealing temperature from 60 °C to 45 °C (−1 °C per cycle) during 40 s, with denaturation at 94 °C for 40 s and extension at 72 °C for 1 min. This was followed by 24 additional cycles of 40 s at 94 °C, 40 s at 45 °C, and 40 s at 72 °C, and a final elongation step of 7 min at 72 °C [25]. Amplification success was confirmed by electrophoresis on 2% agarose gel, and amplicons were sequenced by GENEWIZ (Leipzig, Germany). The sequences were edited and aligned using GENEIOUS 7 [50]. Host species were identified by comparing with reference sequences from GenBank, with matches > 98% considered reliable for species assignment.

### Statistical analyses

All statistical analyses were conducted in R software version 4.3.2 (R Core Development Team, 2020) using the package lme4 [52] and vegan [53]. Although mosquitoes were collected, the number of individuals was insufficient for meaningful statistical analyses. Consequently, mosquito data were excluded from inferential analyses, and all statistical analyses were conducted exclusively using *Culicoides* data.

#### Diversity and abundance of *Culicoides* and host vector choice

We calculated for each habitat type: (i) the species richness, defined as the total number of *Culicoides* species captured; (ii) *Culicoides* diversity, estimated using both the Simpson (D) and the Shannon (H') indices; and (iii) total abundance of *Culicoides*.

Differences in species richness and diversity across habitats were assessed using linear models (LMs) and analysis of variance (ANOVA), treating habitat type as a categorical independent variable with four levels: forest, border, plantation, and village. Prior to ANOVA, we verified assumptions of normality (Shapiro–Wilk test) and homogeneity of variances (Levene’s test), which were satisfied in all cases (*P* > 0.05). To ensure robustness of the results and account for potential deviations from parametric assumptions, we additionally performed nonparametric Kruskal–Wallis tests.

Differences in species composition among habitat types were assessed using nonmetric multidimensional scaling (NMDS) and permutational multivariate analysis of variance (PERMANOVA). We also conducted parallel analyses grouping habitats into two broader categories: natural and anthropogenic areas.

We used linear mixed models (LMMs) to analyze variations in *Culicoides* abundance (both sexes combined, as well as separated by sex) across habitat types. Abundance data were log-transformed to meet normality assumptions. Trap identity was included as random factor, while habitat was a fixed effect. Significance of fixed effects was assessed using likelihood ratio tests, and post hoc comparison tests were performed to examine differences between habitat types. Similar LMMs were also conducted for the natural versus anthropogenic habitat classification.

Additionally, we used LMMs to assess the interaction between habitat type and host-feeding behavior, which we classified as ornithophilic (more than 75% of blood meals from birds) or mammophilic (more than 75% of blood meals from mammals), with trap identity included as a random factor.

#### Parasites screening in *Culicoides*

Haemosporidian infection rates in *Culicoides* were estimated by maximum likelihood (MLE), accounting for pool size. Infection rates are expressed as the estimated number of infected *Culicoides* per 100 individuals (i.e., MLE × 100), with mean values and 95% confidence intervals calculated using the PoolPrev function in the R package PoolTestR [54].

#### Parasites screening in birds

Differences in bird infection status were analyzed using generalized linear mixed models (GLMMs) with a binomial error distribution and a logit link function, treating habitat type (with three levels: forest, border, and oil palm plantation) and season (dry or rainy) as fixed effects. Infection status was treated as a binomial response variable (infected or uninfected), analyzed both for all haemosporidian genera combined and separately for *Plasmodium*, *Haemoproteus*, and *Leucocytozoon*. Bird species nested within their taxonomic family was included as a random effect. Fixed effects were tested using likelihood ratio tests. Individuals showing co-infections that prevented unambiguous genus- or lineage-level identification (*n *= 4) were excluded from the analyses.

## Results

### Insect vectors sampling

Both biting midges (*Culicoides spp*.) and mosquitoes were collected; however no black flies were captured. A total of 40 mosquito specimens representing eight species were recorded (Additional file 1: Table S3). Most mosquitoes were captured in the village (*N* = 23), followed by the oil palm plantation (*N* = 6), border (*N* = 6), and forest (*N* = 5). Mosquito diversity was highest in the village, with seven species identified (*Aedes albopictus*, *Anopheles gambiae*, *Culex* (*Culiciomyia*) *cambournaci*, *Culex micolo*, *Culex decens*, *Uranotenia* (*Pseudoficalbia*) *micromelas*, *Uranotenia* (*Uranotenia*) *connali*), with the *Anopheles gambiae* complex being the most abundant. Due to the low number of specimens and the absence of engorged individuals, mosquitoes were not analyzed further, and subsequent analyses focused exclusively on *Culicoides* biting midges.

### Diversity of *Culicoides* species across habitats

Nine *Culicoides* species were recorded across all habitat types (Table 1). Five species were captured in the forest, seven in the border, five in the oil palm plantation, and all nine in the village (Table 1). Species richness did not differ significantly among habitats (*F* = 2.73; *P* = 0.14), nor did diversity indices (Shannon: *F* = 0.13, *P* = 0.94; Simpson: *F* = 0.06, *P* = 0.98). Kruskal–Wallis tests confirmed these results (Shannon: *P* = 0.92; Simpson: *P* = 0.98). Similarly, species composition did not differ significantly among habitats (*F* = 1.68; *P* = 0.09). Results using the binary classification of habitats (natural versus anthropogenic) are provided in Additional file 2: Tables S4, S5, S6 and Fig. S1.

**Table 1:** Abundance of female *Culicoides* spp. sampled across different habitats on São Tomé Island

Species	Forest	Border	Oil palm plantation	Village	Total individuals	Relative abundance (%)
*C. citroneus*	942 (41.94)	331 (7.28)	24 (5.13)	5 (0.33)	1302	14.79
*C. distinctipennis*	2 (0.09)	10 (0.22)	14 (2.99)	2 (0.13)	28	0.32
*C. hortensis*	873 (38.87)	3399 (74.74)	350 (74.79)	763 (49.55)	5385	61.18
*C. krameri*	0	1 (0.02)	0	74 (4.81)	75	0.85
Neavei group	0	0	0	7 (0.46)	7	0.08
*C. nigripennis*	425 (18.92)	680 (14.95)	28 (5.98)	5 (0.33)	1138	12.93
*C. quinquelineatus*	0	2 (0.04)	0	641 (41.62)	643	7.31
*C. subschultzei*	0	0	0	8 (0.52)	8	0.09
*C. sp. #20*	4 (0.18)	125 (2.75)	52 (11.11)	35 (2.27)	216	2.45
Total	2246	4548	468	1540	8802	

Values represent raw counts, with percentages in parentheses indicating the proportion within each habitat. The last two columns show the total number of individuals per species and their relative abundance in percentage across all habitats

### Abundance and distribution of *Culicoides* species

All traps yielded *Culicoides*. A subset of specimens from each trap was analyzed, resulting in a total count of 11,090 *Culicoides* specimens, representing 9 species. Females accounted for 79.4% of the total catch. *Culicoides hortensis* was the most abundant species, detected across all habitats, while members of the Neavei group were the least abundant, restricted to the village (Table 1). *Culicoides* abundance varied significantly among species (*χ*^2^ = 79.40; *P* < 0.01) and habitats (*χ*^2^ = 8.77; *P* = 0.03). Abundance was significantly lower in the oil palm plantation than in the border (estimate = 1.12; SE 0.44; *P* = 0.01) and forest (estimate = 1.35; SE 0.54; *P* = 0.04). Similar patterns were observed for males, with significant variation across species (*χ*^2^ = 83.27; *P* < 0.01) and habitats (*χ*^2^ = 9.95; *P* = 0.02). Male *Culicoides* were significantly less abundant in the oil palm plantation than in the border (estimate = 1.21; SE 0.41; *P* < 0.01), forest (estimate = 1.36; SE 0.51; *P* < 0.01), and village (estimate = 1.01; SE 0.50; *P* < 0.05). For females only, abundance differed among species (*χ*^2^ = 78.55; *P* < 0.01), while habitat effects were marginal (*χ*^2^ = 6.85; *P* = 0.08). Nonetheless, pairwise comparisons showed significantly lower female *Culicoides* abundant in the oil palm plantation than in the border (estimate = 1.00; SE 0.43; *P* = 0.02), and forest (estimate = 1.14; SE 0.53; *P* = 0.03). Results from the models using the binary classification of habitats (natural versus anthropogenic) are available in the Additional file 2: Table S7.

### Vector host preference

**Fig 4:**
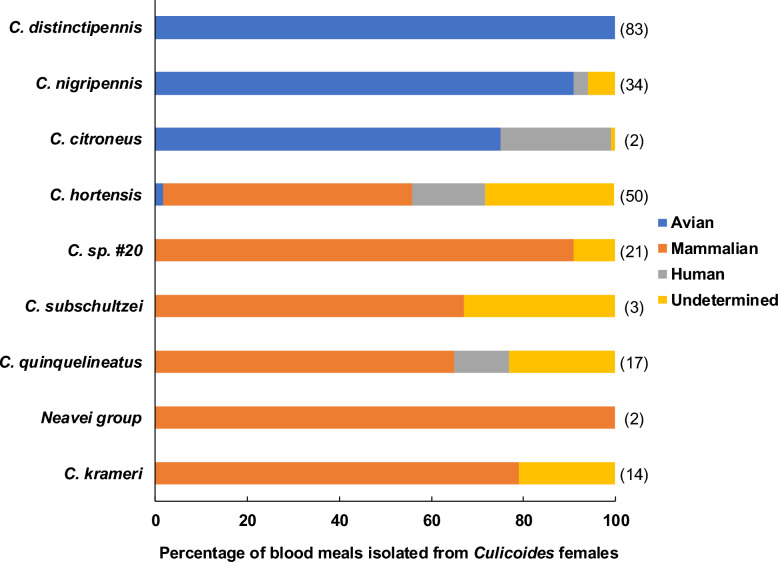
Percentage of blood meals from *Culicoides* species from São Tomé Island. Numbers in the brackets on the right represent the number of individuals analyzed

Blood meals were analyzed for 291 engorged *Culicoides* individuals from 9 species, with host identification successful for 198 individuals (66%), representing 11 different vertebrate host species. Most blood meals were derived from the São Tomé thrush (*Turdus olivaceofuscus*, *N* = 92) and pigs (*Sus scrofa*, *N* = 53; Fig. 4). Mixed blood meals were detected in 15 individuals.

Three *Culicoides* species (*C. distinctipennis*, *C. nigripennis*, and *C. citroneus*) exhibited a predominant preference for avian hosts, while six species (*C. hortensis*, *C. sp*. #20, *C. krameri*, *Neavei group*, *C. quinquelineatus*, and *C. subschultzei*) mainly fed on mammals. Some species exhibited habitat-dependent feeding plasticity. For instance, *C. citroneus*, primarily ornithophilic in forested areas, was also recorded feeding on humans in the plantation. Similarly, *C. hortensis*, mainly mammophilic (including human hosts), was once recorded feeding on a bird (*Gallus gallus*) in the village.

**Fig 5:**
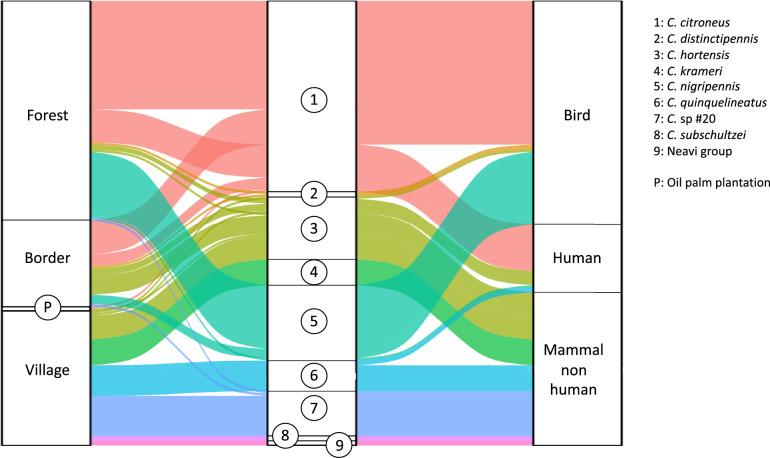
Alluvial graph illustrating the ecological relationships between habitat types, *Culicoides* species, and their feeding behavior on São Tomé Island. The width of the bands connecting categories reflects the relative number of observations across the network

Mammophilic *Culicoides* were significantly more abundant in the village, whereas ornithophilic individuals were more abundant in the forest (estimate = 1.96; SE 0.90; *P* = 0.03). In border and plantation habitats, both feeding behaviors were observed. Both ornithophilic and mammophilic species were recorded, with *C. citroneus* and *C. distinctipennis* feeding on birds, and *C. hortensis* feeding on mammals, although *Culicoides* abundance in the plantation was lower than in the other habitats (Fig. 5).

### Parasite screening in *Culicoides*

Haemosporidian screening was performed on 452 parous *Culicoides* from 7 species, grouped into 105 pools (mean = 4.3 individuals/pool; range 1–10; Table 2 and Additional file 1: Table S1). Parasite were detected in 12 pools from 5 species. *Haemoproteus pastoris* (lineage LAMPUR01) was isolated from a pool of *C. distinctipennis* from the border. *Plasmodium* sp. (lineage COLL7) was found in six pools, involving *C. citroneus* and *C. nigripennis*, from the forest and border. *Plasmodium sp.* (lineage TUROLI01) was detected in two pools of *C. distinctipennis* from the border, as well as in one pool of *C. hortensis* and one pool of *C. quinquelineatus* from the village. A *Leucocytozoon* sp. was isolated from a pool of *C. citroneus* from the border. No infected pools were detected within the plantation, as no parous *Culicoides* were collected there. The border habitat type showed the highest number of positive pools for haemosporidians (Table 2). The minimum infection rates (MLE) were 0.95% for *Haemoproteus*, 0.95% for *Leucocytozoon*, and 9.5% for *Plasmodium.*

**Table 2:** Number of *Culicoides* individuals by pools for haemosporidian parasites screening on São Tomé Island

Biting midges species	Total specimens	Range per pool	Pools analyzed	Positive pools	Infection rate	CILow 95%	CIHigh 95%	Origin of positive samples
*C. citroneus*	178	[1–6]	44	4	2.3	0.7	5.3	Forest (1)
								Border (3)
*C. disctinctipennis*	47	[1–5]	15	3	7	1.8	17.3	Border (3)
*C. hortensis*	103	[1–10]	15	1	1	0.58	4.4	Village (1)
*C. krameri*	35	[5–10]	4	0	0	0	5	
*C. nigripennis*	46	[1–5]	15	3	7	1.8	17.2	Forest (1)
								Border (2)
*C. quinquelineatus*	19	[2–10]	3	1	6.4	0.4	25.8	Village (1)
*C. sp*. #20	24	[1–6]	9	0	0	0	7.7	

Infection rates per species are given with confidence intervals at 95%. In brackets are the number of individuals per pool

### Parasites screening in birds

A total of 432 avian blood samples from 7 species were analyzed. Of these, 25.93% (112/432) were captured in forest areas, 48.38% (209/432) in the border, and 25.69% (111/432) in the oil palm plantation. By season, 45.60% (197/432) were captured in the dry season and 54.40% (235/432) in the rainy season.

Haemosporidian infections were detected in 40.97% of birds (177/432). Among infected birds, 3.39% (6/177) were infected by *Haemoproteus* spp., 27.68% (49/177) by *Leucocytozoon* spp., and 68.93% (122/177) by *Plasmodium* spp. (Table 3). Molecular characterization of haemosporidian infections revealed a total of 25 distinct lineages, including 4 *Haemoproteus*, 12 *Leucocytozoon*, and 9 *Plasmodium* and lineages. *Haemoproteus* infections included the lineages CRIRUF01 (*N* = 3), ZOSLUG02 (*N* = 1), PLOGRA01 (*N* = 1), and ZOSLUG01 (*N* = 1). *Plasmodium* infections comprised the lineages TUROLI01 (*N* = 39), COLL7 (*N* = 39), TUROLI04 (N = 12), MALNI02 (*N* = 9), TUROLI12 (*N* = 8), PLOCUC02 (*N* = 5), WW3 (*N* = 4), PLOPRI01 (*N* = 3), and AFR10 (*N* = 1). Two additional *Plasmodium*-positive samples could not be assigned to a known lineage and were classified as *Plasmodium* sp. *Leucocytozoon* infections showed the highest lineage diversity, with 12 lineages detected, dominated by PLOSAN01 (*N* = 18) and CRIRUF02 (*N* = 12), followed by PLOSAN04 (*N* = 6), PLOSAN02 (*N* = 4), AFR204 (*N* = 2), and several lineages detected at low frequency (*N* = 1): TUROLI10, TUROLI08, AFR117, ZOSLUG05, PLOSAN03, AFR214, and CYAOLI18.

**Table 3:** Haemosporidian parasites across different habitats on São Tomé Island

Parasite	Habitat	*N*	*N* infected	Prevalence (%)
*Haemoproteus*	Forest	112	1	0.89
	Border	209	4	1.91
	Oil palm plantation	111	1	0.9
	Total	432	6	1.39
*Plasmodium*	Forest	112	18	16.07
	Border	209	53	25.36
	Oil palm plantation	111	51	45.95
	Total	432	122	28.24
*Leucocytozoon*	Forest	112	17	15.18
	Border	209	32	15.31
	Oil palm plantation	111	0	0
	Total	432	49	11.34

*N* number of screened birds, *N infected* number of infected birds

Neither habitat nor season affected overall haemosporidian prevalence (respectively: *χ*^2^ = 1.62; *P* = 0.44; estimate = 0.31; SE 0.28; *P* = 0.27). When analyzing by genus, *Plasmodium* prevalence was significantly higher in birds from the oil palm plantation than from the border (estimate = 0.60; SE 0.28; *P* = 0.04) and forest (estimate = 1.02; SE 0.43; *P* = 0.02), with no seasonal effect (estimate = 0.42; SE 0.31; *P* = 0.17). *Haemoproteus* prevalence did not differ between habitats (border versus plantation: estimate = 0.66; SE 1.85; *P* = 0.72, border versus forest: estimate = 0.45; SE 2.16; *P* = 0.83, plantation versus forest: estimate = 0.20; SE 2.06; *P* = 0.92) nor seasons (estimate = 0.21; SE 0.47; *P* = 0.66). *Leucocytozoon* infections were not detected in birds captured in the oil palm plantation, and did not differ between the birds captured in the border and forest (estimate = 0.42; SE 0.64; *P* = 0.51), nor between seasons (estimate = 0.19; SE 0.41; *P* = 0.64). Results from the binary classification of habitats (natural versus anthropogenic) are available in the Additional file 2: Table S8.

## Discussion

Our findings highlight the complex interactions among avian haemosporidian parasites, their avian hosts, and their vectors under anthropogenic disturbances such as deforestation and agricultural expansion. Land-use changes significantly affect vector abundance, host-feeding preference, and parasite prevalence, altering the ecology and transmission dynamics of vector-borne diseases.

### Abundance, diversity, richness, and composition of vectors

We observed significant variation in the abundance of *Culicoides* species across different land uses, with *C. hortensis* being the most abundant and widespread species, while others, such as *Culicoides* of the Neavei group, were present only in the village. The lower abundance of *Culicoides* in the plantation suggests that this habitat is less suitable, likely due to microclimate conditions, the availability of suitable egg-laying substrates, and low availability of preferred vertebrate hosts [55, 56]. Similar patterns have been reported in central Panama, where *Culicoides* were abundant in forested habitats compared with disturbed areas [21], reinforcing the preference of these vectors for more natural environments. Although vector diversity did not differ significantly among habitats, species composition varied between natural and anthropogenic areas (Additional file 2: Table S6 and Fig. S1), potentially reflecting differences in host availability [57, 58].

The low number of mosquitoes captured may be attributed to the traps used, which did not include CO_2_ or olfactory, essential for efficient mosquito sampling [59]. Despite São Tomé hosting high mosquito diversity [60], mosquito abundance in our study area was lower than reported for urban and periurban environments [61]. Previous studies have shown that anthropogenic habitats on São Tomé support higher mosquito richness and density, particularly of species such as *Aedes albopictus*, *Anopheles coluzzii*, and *Culex spp*., probably due to the abundance of artificial breeding sites and host availability [61, 62]. In contrast, forested areas harbor fewer mosquitoes and higher proportion of other Diptera families [24, 34, 60]. The predominance of anthropophilic mosquitoes in human-modified habitats has important epidemiological implications, increasing the risk of vector-borne disease transmission.

### *Culicoides* host-feeding preferences

Distinct host preferences among the *Culicoides* species analyzed, where some species, such as *C. distinctipennis* or *C. nigripennis*, predominantly fed on birds, while others, such as *C. quinquelineatus* or *C. krameri*, fed on mammals, could influence the parasite transmission dynamics [63]. Bird-feeding *Culicoides* were more abundant in forest and border areas, while mammal-feeding species dominated plantations and villages, reflecting differences in host availability across habitats.

On oceanic islands such as São Tomé, native terrestrial vertebrate communities are largely composed of birds, and very few rodents, such as *Crocidura thomensis*, being the only native mammals. Most mammal species present on the island (e.g., livestock, pets, and invasive rodents) have been introduced by humans and are concentrated in disturbed habitats. Consequently, primary forests are expected to sustain a higher abundance of ornithophilic vectors, whereas mammal-associated vectors are more prevalent in anthropogenic environments. This pattern likely explains the observed distribution of *Culicoides* feeding guilds across land uses.

The predominance of mammal-feeding *Culicoides* in human-modified landscapes may also enhance the transmission of pathogens affecting humans and domestic animals. For example, in Europe, *Culicoides imicola*, the main vector of Bluetongue virus, thrives in areas with high livestock densities [64]. Other pathogens transmitted by *Culicoides* include Oropouche virus in South America [65] and the filarial worm *Mansonella perstans* in West Africa [66], highlighting the need for further surveillance of pathogens carried by *Culicoides* species in São Tomé.

### Parasite screening in *Culicoides*

The haemosporidian screening in *Culicoides* suggests that *C. distinctipennis* could play a pivotal role as a primary vector for *Haemoproteus* parasites, while *C. citroneus* and *C. nigripennis* could act as secondary vectors. The detection of *Plasmodium* and *Leucocytozoon* lineages, transmitted by mosquitoes [67] and black flies [68], respectively, in *Culicoides* pools suggests potential abortive infection stages, where the parasite fails to complete its life cycle in the vector [35]. Similarly, previous studies have also reported *Plasmodium* and *Leucocytozoon* parasites in parous *Culicoides* [69–71].

### Parasite infections in birds

All the lineages found in our study were isolated before on the island [32]. The haemosporidian screening in avian blood revealed significant differences in parasite distribution and infection prevalence across habitats. These habitat-specific differences likely reflect the ecological requirements of their respective vectors. *Plasmodium* prevalence was higher in disturbed habitats, supporting the hypothesis that these environments enhance mosquito proliferation, and consequently, *Plasmodium* transmission [72]. In fact, although the number of mosquitoes captured during this study was low, their highest abundance was observed in disturbed habitats, aligning with this pattern. In contrast, *Leucocytozoon* prevalence was absent in birds from oil palm plantations, likely due to the lack of suitable habitats for black fly vectors, which require clear, flowing water typically found in forested areas [73]. This could explain the higher prevalence of *Leucocytozoon* infections in birds from forest areas, as also observed in other recent studies [32, 74]. These results highlight how vector ecology is closely linked to land-use patterns, shaping the spatial distribution of parasite infections. Similarly, in Cameroon, deforestation significantly influenced the prevalence of avian *Plasmodium* and *Haemoproteus* parasites. Consistent with our results, they observed the highest prevalence of *Haemoproteus* infections in the forests. However, contrary to our findings, they reported the highest prevalence of *Plasmodium* infections in pristine forest and the lowest prevalence in young oil palm plantations [14]. The lack of significant differences in *Haemoproteus* prevalence across habitats, despite the overall low infection rates of this genus, suggests that other factors, such as the distribution of *Culicoides* and their feeding behavior, may be modulating the transmission dynamics of these parasites.

## Conclusions

Our study reinforces evidence that land-use changes, particularly deforestation and agricultural expansion, strongly influences vector communities and pathogen transmission dynamics. Disturbed habitats may increase transmission potential although outcomes vary among systems. Understanding these dynamics require integrative approaches that combine vector ecology, host community structure, and environmental change. Future studies incorporating temporal variation, functional traits, and experimental approaches will improve predictions of vector-borne disease responses to land-use change.

## Supplementary Information

Additional file 1.

Additional file 2.

## Data Availability

Data supporting the main conclusions of this study are included in the manuscript.
