# Supplementary material for: Oil palm expansion reshapes Culicoides assemblages and avian haemosporidian infections
Source: Parasit Vectors. 2026 Mar 10;19:139. doi: 10.1186/s13071-026-07319-y (PMC13036970; doi:10.1186/s13071-026-07319-y)
Supplement: Supplementary file 1 — Additional file 1. [file 13071_2026_7319_MOESM1_ESM.docx]

**Additional file 1**

**From forest to oil palm plantation: Impact of land-use changes on insect-vector communities, host preferences and parasite prevalence**

Rafael Gutiérrez-López, Bruno Mathieu, Boris K. Makanga, Christophe Paupy, Nil Rahola, Vincent Bourret, Martim Melo, Claire Loiseau

**Table S1**. Summary of *Culicoides* pools used for haemosporidian screening by habitat category and species. (Num_Ind_Pool: number of individuals per pool).

| **ID_Pool** | **Numi_Ind_Pool** | **Specie** | **Habitat** | **Results** | **Parasite** |
| --- | --- | --- | --- | --- | --- |
| P1 | 1 | *C. citroneus* | Forest | 0 |  |
| P2 | 3 | *C. citroneus* | Border | 0 |  |
| P3 | 5 | *C. citroneus* | Forest | 0 |  |
| P4 | 1 | *C. citroneus* | Forest | 0 |  |
| P5 | 3 | *C. citroneus* | Forest | 0 |  |
| P6 | 5 | *C. citroneus* | Border | 0 |  |
| P7 | 5 | *C. citroneus* | Forest | 0 |  |
| P8 | 5 | *C. citroneus* | Forest | 0 |  |
| P9 | 5 | *C. citroneus* | Forest | 0 |  |
| P10 | 6 | *C. hortensis* | Forest | 0 |  |
| P11 | 2 | *C. disctinctipennis* | Forest | 0 |  |
| P12 | 3 | *C. nigripennis* | Forest | 0 |  |
| P13 | 6 | *C.* sp. #20 | Forest | 0 |  |
| P14 | 4 | *C. citroneus* | Border | 0 |  |
| P15 | 4 | *C. citroneus* | Border | 0 |  |
| P16 | 5 | *C. hortensis* | Border | 0 |  |
| P17 | 3 | *C. disctinctipennis* | Border | 1 | *Haemoproteus* Lampur01 |
| P18 | 5 | *C. nigripennis* | Border | 1 | *Plasmodium* Coll7 |
| P19 | 3 | *C. nigripennis* | Border | 0 |  |
| P20 | 2 | *C.* sp. #20 | Border | 0 |  |
| P21 | 5 | *C. citroneus* | Border | 1 | *Leucocytozoon* sp. |
| P22 | 5 | *C. citroneus* | Border | 0 |  |
| P23 | 8 | *C. hortensis* | Border | 0 |  |
| P24 | 1 | *C. disctinctipennis* | Border | 0 |  |
| P25 | 3 | *C. nigripennis* | Border | 0 |  |
| P26 | 3 | *C.* sp. #20 | Border | 0 |  |
| P27 | 5 | *C. citroneus* | Border | 0 |  |
| P28 | 1 | *C. citroneus* | Border | 0 |  |
| P29 | 5 | *C. hortensis* | Border | 0 |  |
| P30 | 2 | *C. disctinctipennis* | Border | 0 |  |
| P31 | 2 | *C. nigripennis* | Border | 0 |  |
| P32 | 2 | *C.* sp. #20 | Border | 0 |  |
| p33 | 5 | *C. citroneus* | Forest | 0 |  |
| p34 | 5 | *C. citroneus* | Forest | 0 |  |
| p35 | 2 | *C. citroneus* | Forest | 0 |  |
| p36 | 10 | *C. hortensis* | Forest | 0 |  |
| p37 | 4 | *C. hortensis* | Forest | 0 |  |
| P38 | 5 | *C. disctinctipennis* | Forest | 0 |  |
| P39 | 1 | *C. disctinctipennis* | Forest | 0 |  |
| P40 | 5 | *C. nigripennis* | Forest | 0 |  |
| P41 | 2 | *C.* sp. #20 | Forest | 0 |  |
| P42 | 5 | *C. citroneus* | Border | 0 |  |
| P43 | 5 | *C. citroneus* | Border | 0 |  |
| P44 | 5 | *C. citroneus* | Border | 0 |  |
| P45 | 6 | *C. citroneus* | Border | 0 |  |
| P46 | 7 | *C. hortensis* | Border | 0 |  |
| P47 | 5 | *C. disctinctipennis* | Border | 0 |  |
| P48 | 5 | *C. disctinctipennis* | Border | 0 |  |
| P49 | 5 | *C. nigripennis* | Border | 0 |  |
| P50 | 3 | *C. nigripennis* | Border | 0 |  |
| P51 | 3 | *C. nigripennis* | Border | 0 |  |
| P52 | 1 | *C.* sp. #20 | Border | 0 |  |
| P53 | 5 | *C. citroneus* | Border | 0 |  |
| P54 | 4 | *C. citroneus* | Border | 0 |  |
| P55 | 4 | *C. hortensis* | Border | 0 |  |
| P56 | 5 | *C. disctinctipennis* | Border | 0 |  |
| P57 | 2 | *C. disctinctipennis* | Border | 0 |  |
| P58 | 1 | *C.* sp. #20 | Border | 0 |  |
| P59 | 5 | *C. citroneus* | Border | 0 |  |
| P60 | 1 | *C. hortensis* | Border | 0 |  |
| P61 | 4 | *C. disctinctipennis* | Border | 1 | *Plasmodium* TUROLi01 |
| P62 | 4 | *C. disctinctipennis* | Border | 1 | *Plasmodium* TUROLi01 |
| P63 | 4 | *C. disctinctipennis* | Border | 0 |  |
| P64 | 1 | *C. nigripennis* | Border | 0 |  |
| P65 | 3 | *C.* sp. #20 | Border | 0 |  |
| P66 | 4 | *C. nigripennis* | Forest | 0 |  |
| P67 | 5 | *C. citroneus* | Forest | 0 |  |
| P68 | 5 | *C. citroneus* | Forest | 0 |  |
| P69 | 6 | *C. citroneus* | Forest | 0 |  |
| P70 | 2 | *C. nigripennis* | Forest | 1 | *Plasmodium* Coll7 |
| p71 | 5 | *C. citroneus* | Forest | 0 |  |
| P72 | 2 | *C. citroneus* | Forest | 1 | *Plasmodium* Coll7 |
| P73 | 3 | *C. citroneus* | Forest | 0 |  |
| P74 | 3 | *C. nigripennis* | Forest | 0 |  |
| P75 | 5 | *C. citroneus* | Forest | 0 |  |
| P76 | 5 | *C. citroneus* | Forest | 0 |  |
| P77 | 5 | *C. citroneus* | Forest | 0 |  |
| P78 | 5 | *C. citroneus* | Forest | 0 |  |
| P79 | 4 | *C. citroneus* | Forest | 0 |  |
| P80 | 2 | *C. citroneus* | Forest | 0 |  |
| P81 | 2 | *C. nigripennis* | Border | 0 |  |
| P82 | 2 | *C. citroneus* | Border | 0 |  |
| P83 | 2 | *C. citroneus* | Border | 0 |  |
| P84 | 2 | *C. nigripennis* | Border | 1 | *Plasmodium* Coll7 |
| P85 | 4 | *C. citroneus* | Border | 1 | *Plasmodium* Coll7 |
| P86 | 3 | *C. citroneus* | Border | 1 | *Plasmodium* Coll7 |
| p87 | 3 | *C. hortensis* | Border | 0 |  |
| P88 | 3 | *C. citroneus* | Border | 0 |  |
| P89 | 4 | *C. citroneus* | Border | 0 |  |
| P90 | 1 | *C. disctinctipennis* | Border | 0 |  |
| P91 | 4 | *C. citroneus* | Forest | 0 |  |
| P92 | 10 | *C. hortensis* | Village | 0 |  |
| P93 | 4 | *C.* sp. #20 | Village | 0 |  |
| P94 | 2 | *C. quinquelineatus* | Village | 0 |  |
| P95 | 10 | *C. hortensis* | Village | 0 |  |
| P96 | 10 | *C. hortensis* | Village | 1 | *Plasmodium* TUROLi01 |
| P97 | 10 | *C. hortensis* | Village | 0 |  |
| P98 | 10 | *C. hortensis* | Village | 0 |  |
| P99 | 10 | *C. quinquelineatus* | Village | 0 |  |
| P100 | 7 | *C. quinquelineatus* | Village | 1 | *Plasmodium* TUROLi01 |
| P101 | 10 | *C. krameri* | Village | 0 |  |
| P102 | 10 | *C. krameri* | Village | 0 |  |
| P103 | 10 | *C. krameri* | Village | 0 |  |
| P104 | 5 | *C. krameri* | Village | 0 |  |
| P105 | 3 | *C. disctinctipennis* | Village | 0 |  |

**Table S2**. Sampling sites on São Tomé Island sorted by land use type (plantation, border and forest), year(s) of blood collection and coordinates.

| **Habitat** | **Sampling site** | **Coordinates** | **Year** |
| --- | --- | --- | --- |
| Plantation | Martim Mendes Bridge | 0°07'38.0"N 6°35'30.0"E | 2018-2019 |
| Plantation | Vila Zé | 0°07'07.0"N 6°35'12.0"E | 2019 |
| Plantation | Mateus Sampaio (village) | 0°06'51.0"N 6°35'42.0"E | 2019 |
| Border | Agostinho Base | 0°08'14.4"N 6°34'37.5"E | 2018-2019 |
| Border | Monte Carmo Low Camp | 0°08'20.0"N 6°34'33.0"E | 2018-2019 |
| Forest | Monte Carmo Above | 0°08'28.0"N 6°34'19.0"E | 2019 |
| Forest | Umbumgu | 0°09'13.0"N 6°33'49.0"E | 2016 |

**Table S3**. Number of mosquito specimens (N) collected by species and habitat type. Species found in multiple habitats are listed with corresponding counts per habitat.

| **Mosquito species** | **Habitat** | **N** |
| --- | --- | --- |
| *Aedes albopictus* | Village | 1 |
| *Anopheles coustani* | Plantation | 1 |
| *Anopheles gambiae* | Village | 16 |
| *Culex cambournaci* | Forest | 1 |
|  | Border | 3 |
|  | Plantation | 1 |
|  | Village | 8 |
| *Culex decens* | Village | 7 |
| *Culex micolo* | Village | 2 |
| *Culex sp* | Forest | 1 |
|  | Village | 2 |
| *Lutzia tigripes* | Border | 1 |
| *Uranotenia connali* | Village | 9 |
| *Uranotenia micromelas* | Forest | 4 |
|  | Border | 5 |
|  | Plantation | 4 |
|  | Village | 2 |
| *Uranotenia sp* | Village | 1 |
