# Supplementary material for: Oil palm expansion reshapes Culicoides assemblages and avian haemosporidian infections
Source: Parasit Vectors. 2026 Mar 10;19:139. doi: 10.1186/s13071-026-07319-y (PMC13036970; doi:10.1186/s13071-026-07319-y)
Supplement: Supplementary file 2 — Additional file 2. [file 13071_2026_7319_MOESM2_ESM.docx]

**Additional file 2**

**From forest to oil palm plantation: Impact of land-use changes on insect-vector communities, host preferences and parasite prevalence**

Rafael Gutiérrez-López, Bruno Mathieu, Boris K. Makanga, Christophe Paupy, Nil Rahola, Vincent Bourret, Martim Melo, Claire Loiseau

**Table S4**. Species richness per habitat (alpha-diversity) and ANOVA test results. Results for the land use types defined in two categories (natural vs. anthropogenic areas). The term ‘anthropogenic areas’ includes edges with the core plantation, plantation and village.

| **Analysis** | **Category/Source** | **Species** | **Mean Square** | **F** | **P** |
| --- | --- | --- | --- | --- | --- |
| Species richness per habitat | Disturbed | 10 |  |  |  |
|  | Natural | 7 |  |  |  |
| Species richness vs habitat | Habitat |  | 4.15 | 2.07 | 0.18 |
| (ANOVA) | Residuals |  | 2.00 |  |  |

**Table S5**. Diversity indices *vs* number of species (linear regression models) for the land use types defined in two categories (natural vs. anthropogenic areas). Estimates, standard errors (SE) and p-value (P) are given.

| **Analysis** | **Variable** | **Estimate** | **S.E.** | **P** |
| --- | --- | --- | --- | --- |
| Shannon index | Intercept | 1.98 | 0.63 | 0.01 |
|  | N° species | 0.09 | 0.12 | 0.44 |
| Simpson index | Intercept | 0.78 | 0.20 | <0.01 |
|  | N° species | 0.02 | 0.04 | 0.57 |

**Table S6**. PERMANOVA results based on Bray-curtis distance for the land use types defined in two categories (natural vs. anthropogenic areas). Degrees of freedom (Df), R-squared (R^2^) and p-value (P) are given.

| **Variable** | **Df** | **R²** | **P** |
| --- | --- | --- | --- |
| **Habitat** | **1** | **0.17** | **0.03** |
| Residuals | 12 | 0.83 |  |

**Table S7**. Drivers of the abundance of *Culicoides* in São Tomé Island. Results of the generalized linear mixed models (binomial distribution) for the total, male and females *Culicoides* (log-transformed), with the land use types defined in two categories (natural versus anthropogenic areas). Estimates, standard errors (SE) and p-value (P) are given.

| **Model** | **Variable** | **Estimate** | **S.E.** | **P** |
| --- | --- | --- | --- | --- |
| Total | Intercept | 3.66 | 0.22 | <0.01 |
| total~habitat + (1\|tramp) |  |  |  |  |
|  | **Habitat (anthropogenic)** | **-0.77** | **0.30** | **0.01** |
| Males | Intercept | 2.08 | 0.21 | <0.01 |
| male~habitat + (1\|tramp) |  |  |  |  |
|  | **Habitat (anthropogenic)** | **-0.71** | **0.28** | **0.01** |
| Females | Intercept | 3.40 | 0.22 | <0.01 |
| female~habitat + (1\|tramp) |  |  |  |  |
|  | **Habitat (anthropogenic)** | **-0.67** | **29** | **0.01** |

**Table S8**. Drivers of the abundance of blood parasites in birds from São Tomé Island. Results of the generalized linear mixed models (binomial distribution) for the prevalence of Haemosporidians, *Haemoproteus*, *Plasmodium* and *Leucocytozoon*, with the land use types defined in two categories (natural versus anthropogenic areas) and the season (rainy *vs*. dry). In the latter case, the term ‘anthropogenic areas’ includes edges with the core plantation, plantation and village. Estimates, standard errors (SE) and p-value (P) are given.

| **Model** | **Variable** | **Estimate** | **S.E.** | **P** |
| --- | --- | --- | --- | --- |
| Haemosporidians | (Intercept) | -0.24 | 0.50 | 0.63 |
| Haemosporidians ~ Habitat + Season | **Habitat (anthropogenic)** | **0.55** | **0.26** | **0.04** |
|  | **Season (rainy)** | **-0.54** | **0.25** | **0.03** |
| *Haemoproteus* | (Intercept) | -6.06 | 1.62 | <0.01 |
| *Haemoproteus* ~ Habitat + Season | Habitat (anthropogenic) | 1.14 | 1.43 | 0.42 |
|  | Season (rainy) | 0.59 | 0.96 | 0.53 |
| *Plasmodium* | (Intercept) | -2.23 | 0.82 | <0.01 |
| *Plasmodium* ~ Habitat + Season | **Habitat (anthropogenic)** | **0.65** | **0.28** | **0.02** |
|  | Season (rainy) | -0.51 | 0.27 | 0.06 |
| *Leucocytozoon* | (Intercept) | -1.75 | 0.59 | <0.01 |
| *Leucocytozoon* ~ Habitat + Season | **Habitat (anthropogenic)** | **-2.86** | **0.54** | **<0.01** |
|  | Season (rainy) | 0.19 | 0.41 | 0.64 |

**Fig. S1**. Non-metric multidimensional scaling (NMDS) plot based on Bray-curtis dissimilarities showing the ordination of samples from two habitat types: anthropogenized (orange) and natural (blue). Each point represents a sample, and proximity between points indicates similarity in species composition.
